# Impact of extreme (flat and steep) keratometry on the safety and efficacy of small incision lenticule extraction (SMILE)

**DOI:** 10.1038/s41598-021-97375-4

**Published:** 2021-09-08

**Authors:** Nikolaus Luft, Jakob Siedlecki, Franziska Reinking, Wolfgang J. Mayer, Benedikt Schworm, Stefan Kassumeh, Siegfried G. Priglinger, Martin Dirisamer

**Affiliations:** 1 Department of Ophthalmology, Ludwig-Maximilians-University, Mathildenstrasse 8, 80336 Munich, Germany; 2 SMILE Eyes Clinic, Linz, Austria

**Keywords:** Outcomes research, Surgery

## Abstract

Little is known about the connection between preoperative keratometry and postoperative results of myopic small-incision lenticule extraction (SMILE). To determine the influence of extreme (flat and steep) corneal keratometry on the safety and efficacy of SMILE, the databases of the Department of Ophthalmology, Ludwig-Maximilians-University Munich, Germany, and SMILE Eyes Linz, Austria, were screened for patients with steep and flat keratometry who had undergone SMILE. In this cross-sectional matched comparative cohort study, eyes with markedly flat (< 42.0 diopters; D) or steep (≥ 47.0D) preoperative corneal keratometry were matched to a cohort of eyes with regular keratometry (42.0–46.9D) by preoperative manifest refractive spherical equivalent and cylinder, age, corrected distance visual acuity and surgical SMILE parameters. The standardized graphs and terms for refractive surgery results were applied to compare the three groups. Changes in higher order aberrations (HOAs) were evaluated on Scheimpflug imaging. In total, 63 eyes (21 each) of 54 patients with a mean refractive spherical equivalent of  − 5.21 ± 1.59 D were followed up for a mean of 9.2 ± 6.1 (minimum ≥ 3) months. Mean baseline keratometry was 41.3 ± 0.7D (flat), 45.5 ± 1.0D (regular) and 47.7 ± 0.6D (steep) (p < 0.0001). Compared to the regular group, the flat and the steep cornea group resulted in a non-inferior percentage of eyes within ± 0.50 D of target refraction (p = 0.20), uncorrected distance visual acuity (p = 0.95) and corrected distance visual acuity (p = 0.20). Flat corneas however experienced a stronger induction of spherical aberration (SA) compared to the steep group (p = 0.0005). In conclusion, non-inferior outcomes of SMILE can also be expected in eyes with steep (≥ 47D) or flat (< 42D) preoperative keratometry, while SMILE however induces more SA in eyes with a flat keratometry.

## Introduction

In the treatment of myopia and myopic astigmatism, small-incision lenticule extraction (SMILE) has become an increasingly popular alternative to laser in situ keratomileusis (LASIK). The former offers on-par results concerning safety and efficacy^1–5^, and probably carries advantages regarding corneal biomechanics^6^, corneal wound healing^7^, and iatrogenic dry eye symptoms^8,9^.

**Figure 1:**
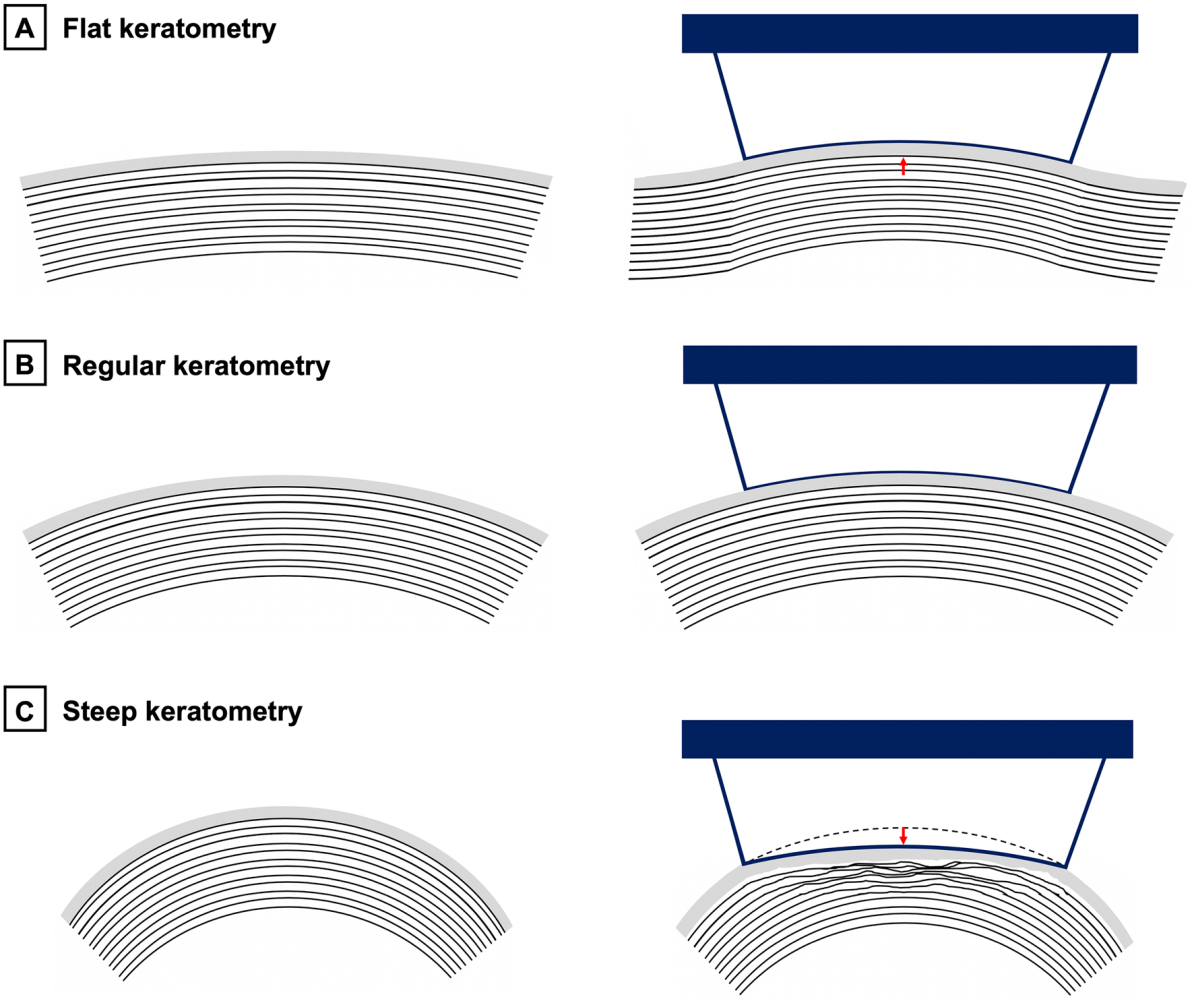
Tissue distortion due to a possible discrepancy between the radius of the femtosecond-laser contact glass and corneal curvature. In flat corneas (**A**), excess suction might deform the stroma by lifting the corneal surface into the contact glass. In steep corneas (**C**), central tissue compression might occur.

To treat myopia or myopic astigmatism, all keratorefractive procedures rely on the flattening of central corneal curvature to produce an “optical zone” with altered corneal power. in excimer-based keratorefractive surgery (LASIK and photorefractive keratometry; PRK), preoperative keratometry can influence postoperative refractive and visual outcomes, especially in extremely flat or steep keratometry, and even more so in conjunction with high myopic ablations^10–13^. SMILE performed with the VisuMax platform (Carl Zeiss Meditec AG, Jena, Germany) requires direct contact with a disposable interface of variable diameter, but uniform radius, which represents a “one-curvature-fits-all” approach. Therefore, in steep corneas, excessive applanation (compression of the central cornea “via the apex”) might cause tissue irregularities within the treated stromal tissue. Moreover, in flat corneas, excess suction may be required to accommodate the corneal surface into the relatively steeper contact glass (Fig. 1). Hence, after femtosecond laser application and release of suction with subsequent relaxation of the corneal tissue irregular cap or lenticule interfaces might emerge.

While there is plenty of literature on the relationship between preoperative keratometry and the outcomes of LASIK and photorefractive keratectomy (PRK)^10–13^, data on SMILE is still lacking. Hence, the objective for this study was to compare the outcomes of myopic SMILE in eyes with excessively steep and flat preoperative keratometry to regular keratometry regarding safety, efficacy and higher order aberrometric outcome.

## Participants and methods

### Participants

For the purpose of this cross-sectional study, the database of the SMILE Eyes Clinic (Linz, Austria) encompassing more than 1800 SMILE procedures was screened for eyes with preoperative flat (< 42D) or steep (≥ 47D) keratometry using the K1 (steeper meridian) value. Within these two groups, eyes were matched concerning preoperative manifest refraction spherical equivalent (MRSE), manifest sphere and cylinder, age, preoperative corrected and uncorrected distance visual acuity (CDVA, UDVA) and surgical SMILE parameters (optical zone, cap thickness). Eyes that did not find a matching partner were removed from analysis. Afterwards, secondary matching to a third group with regular keratometry (42.0–46.9D) was performed based on the same parameters mentioned above. Minimum follow-up was 3 months. Informed consent from each patient as well as Institutional Review Board approval from the Department of Ophthalmology, Ludwig Maximilian’s University was obtained prior to the surgical procedure and data analysis. All study procedures adhered to the tenets of the Declaration of Helsinki and relevant local guidelines and regulations.

### Preoperative assessment

At first presentation, a standardized preoperative protocol was followed as established for all patients undergoing corneal refractive surgery. This included slit lamp-biomicroscopy and fundoscopy, objective, subjective and cycloplegic manifest refraction and Pentacam corneal tomography (Oculus Optikgeräte GmbH, Wetzlar, Germany) to rule out contraindications for keratorefractive treatments (e.g. keratoconus).

### SMILE

SMILE was performed as described previously^1,14–16^ with a standard cap thickness of 120 to 140 µm and an optical zone of 6.4 to 6.7 mm. In brief, surgery was performed under topical anesthesia with oxybuprocaine hydrochloride (Bausch + Lomb, Rochester, NY, USA) using a 500 kHz femtosecond laser (Visumax 500; Carl Zeiss Meditec AG). After sterile draping, the eye of the patient was placed under the femtosecond laser’s operating microscope, centered and immobilized with a suction contact glass. The size “small” was used in all cases. All patients were operated by one of two experienced surgeons (M.D., S.G.P.) using the same laser scanning settings (4.5 µm spot spacing for the cap and lenticule interface at 160 nJ laser energy).

One side cut of 4.00 mm arcuate length was created at the 135° and 45° position in the right and left eyes, respectively. After manual dissection using a blunt spatula, the lenticule was removed through the incision as described previously and checked on the corneal surface for completeness. Then, the interface was irrigated with balanced salt solution. The post-operative treatment regimen consisted of tobramycin/dexamethasone combination eyedrops (Tobradex, Alcon, Hünenburg, Switzerland) six times daily for the first week, dexamethasone (DexaSine, Alcon) eyedrops from the second until the fourth week four times daily, and lubricant eye drops tapered as needed.

### Statistical analysis

All data were gathered and analyzed in Microsoft Excel spreadsheets (Version 15.39 for Mac; Microsoft, Redmond, WA, USA) according to the established graphic reporting of outcomes of refractive surgery^17^. Statistical analysis was performed in SPSS Statistics 23 (SPSS Inc., Chicago*, *IL*, *USA). Normality of data was assessed with the Kolmogorov–Smirnov test. Homogeneity of variance was checked using Levene’s test. Analysis of variance (ANOVA), the Kruskal–Wallis and the Chi-Square test were used to compare the groups as appropriate. The independent samples t-test and Mann–Whitney U test were performed as post-hoc tests with Bonferroni-Holm correction to compensate for multiple testing. Comparing all three groups, the level to indicate statistical significance was defined as p < 0.05; using Bonferroni’s correction, the level of significance was adjusted to p < 0.0167 for post-hoc analyses.

## Results

A total of 63 eyes 54 patients were included in the study, with 21 eyes in each of the flat, regular and steep group. Mean preoperative MRSE was − 5.21 ± 1.59 D and surgical target refraction was plano in all eyes, aside from 2 eyes (9.5%) in both the flat and steep group and 4 eyes (19.0%) in the regular group that were targeted for mini-monovision of up to − 1.0D. The mean postoperative follow-up was 9.2 ± 6.1 months. Patients’ baseline parameters can be found in Table 1. In brief, all groups differed significantly in mean keratometry (flat: 41.3 ± 0.7 vs. regular: 45.4 ± 1.0 vs. steep: 47.7 ± 0.6 D; p < 0.0001). On the contrary, there was no difference concerning mean age (p = 0.22), mean follow-up (p = 0.66), preoperative MRSE (p = 0.94) or manifest cylinder (p = 0.50), CDVA (p = 0.05), pachymetry (p = 0.72), residual stromal bed (p = 0.69) and SMILE treatment parameters (p = 0.56, p = 0.65; Table 1).

**Table 1:** Baseline parameters of the patient cohort.

	Flat	Regular	Steep	Omnibus p-value	Post-hoc F-R	Post-hoc F-S	Post-hoc R-S
Radius (D)	41.3 ± 0.7 (39.7 to 41.9)	45.4 + 1.0 (43.4. to 46.9)	47.7 ± 0.6 (47.0 to 48.9)	< 0.00001	< 0.00001	< 0.00001	< 0.00001
No. of eyes (n)	21	21	21				
No. of patients (n)	15	21	18				
Gender (m/f)	10/5	5/16	2/16				
Mean age (y)	36 ± 8 (26 to 49)	33 + 8 (22 to 49)	32 ± 8 (23 to 52)	0.22	0.08	0.06	0.94
Mean follow-up (m)	10 ± 7 (3 to 36)	7 ± 4 (3 to 33)	8 ± 5 (3 to 18)	0.66	0.09	0.28	0.43
**CDVA** preOP (logMAR)	− 0.01 ± 0.06 (− 0.10 to 0.10)	− 0.05 ± 0.09 (− 0.20 to 0.20)	− 0.01 ± 0.07 (− 0.10 to 0.10)	0.05	0.71	0.83	0.07
Pachymetry (µm)	552 ± 3 (502 to 608)	545 ± 3 (498 to 612)	550 ± 3 (491 to 593)	0.72	0.22	0.43	0.29
Res. Stromal Bed (µm)	322 ± 4 (251 to 403)	312 ± 4 (258 to 385)	315 ± 3 (250 to 378)	0.69	0.22	0.28	0.39
**SMILE**							
Optical Zone (mm)	6.5 ± 0.1 (6.6.50 to 6.70)	6.5 ± 0.0 (6.5 to 6.5)	6.5 ± 0.0 (6.4 to 6.5)	0.56	0.44	0.31	0.80
Cap (µm)	133 ± 8 (120 to 140)	131 ± 9 (110 to 140)	132 ± 8 (120 to 140)	0.65	0.42	0.74	0.65
**MRSE** preOP (D)	− 5.10 ± 1.71 (− 8.06 to − 2.50)	− 5.25 ± 1.56 (− 7.75 to − 2.88)	− 5.26 ± 1.56 (− 7.50 to − 2.50)	0.94	0.39	0.38	0.49
**Manifest cylinder** preOP (D)	− 0.76 ± 0.56 (− 2.25 to 0.00)	− 0.98 ± 0.63 (− 2.00 to 0.00)	− 0.93 ± 0.70 (− 2.25 to 0.00)	0.50	0.28	0.35	0.91

### Safety

**Figure 2:**
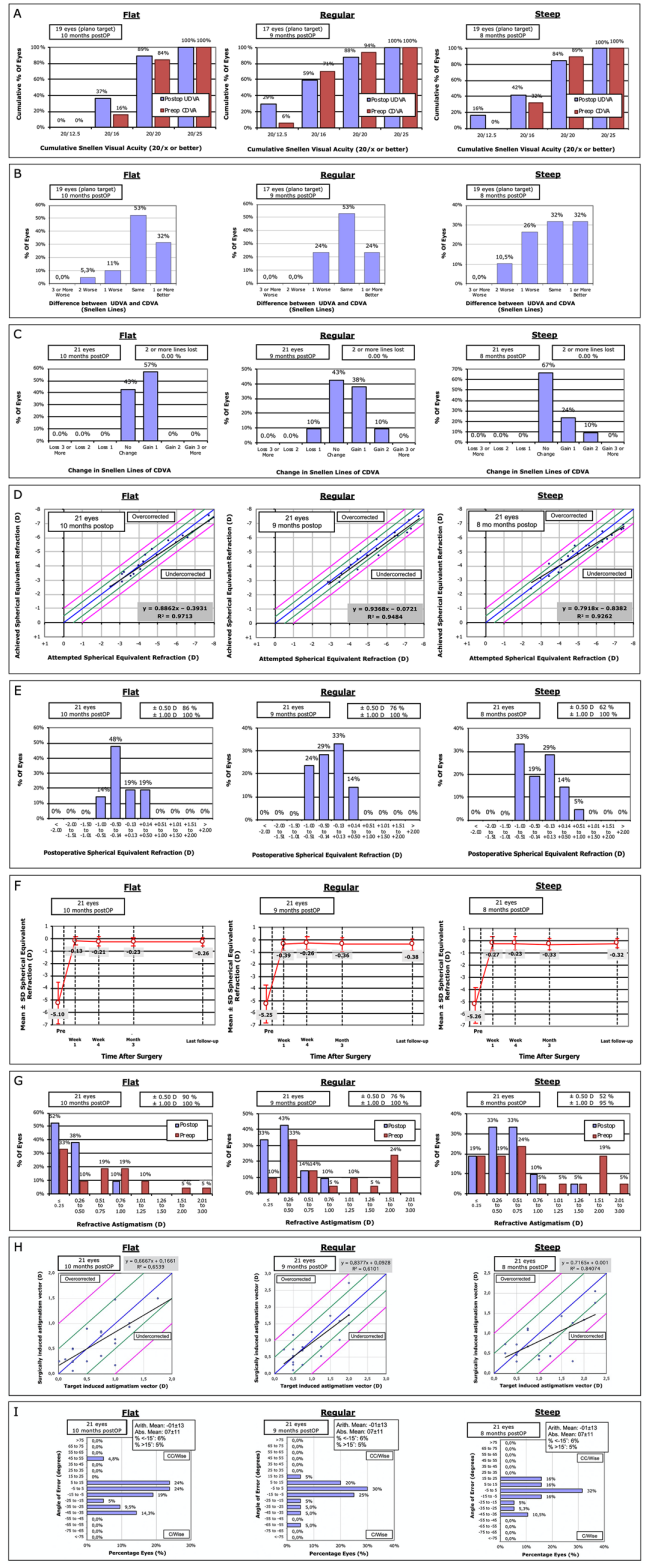
Nine standard graphs for reporting refractive outcomes. (**A**) Efficacy—uncorrected distance visual acuity (UDVA). (**B**) UDVA vs CDVA—comparison of uncorrected to preoperative corrected distance visual acuity. (**C**) Safety (Change in CDVA). (**D**) Attempted vs Achieved Spherical Equivalent Refraction. (**E**) Accuracy of Spherical Equivalent Refraction. (**F**) Stability of Spherical Equivalent Refraction. (**G**) Amplitude of Astigmatism. (**H**) Target induced astigmatism (TIA) vs surgically induced astigmatism (SIA). (**I**) Angle of Error.

There were no intra-, peri- or postoperative complications in either group. There was no loss of two or more lines in any group (Fig. 2). Loss of one line of CDVA was observed in two eyes (10%) in the regular keratometry group only. CDVA at end of follow-up was comparable (flat: − 0.07 ± 0.05 vs. regular: − 0.10 ± 0.10 vs. steep: − 0.06 ± 0.09, p = 0.20). The safety index was similar across all groups (1.13 vs. 1.06 vs. 1.11, p = 0.75).

### Efficacy

There was no difference in refractive accuracy or stability at the end of follow-up (attempted/achieved spherical equivalent: 0.20 ± 0.32 vs. 0.26 ± 0.35 vs. 0.25 ± 0.47; p = 0.40; Table 2; Fig. 2). Moreover, the number of patients within ± 0.50 D of target was comparable between all groups (85.7 vs. 76.2 vs. 61.9%, p = 0.20). All eyes of all groups were within ± 1.0D of target refraction. Regarding manifest cylinder, there was no difference in postoperative values in the flat and steep compared to the regular group (p = 0.24, p = 0.15), but eyes with steep keratometry showed higher postoperative values than eyes with flat keratometry (− 0.62 ± 0.32 vs. − 0.39 ± 0.26, p = 0.02). This also translated into a lower percentage of patients with astigmatism < 0.50 D in the steep as compared to the flat group (52.4% vs. 90.2%; p = 0.006), while the flat and steep group themselves did not show any difference compared to the regular group (p = 0.21, p = 0.11).

**Table 2:** Surgical outcomes of SMILE.

	** Flat **	** Regular **	** Steep **	**omnibus p-value**	**post-hoc F-R**	**post-hoc F-S**	**post-hoc R-S**
Mean SEQ postOP (D)	− 0.26 ± 0.34 (− 0.75 to 0.38; monovision up to − 0.75 D)	− 0.38 ± 0.48 (− 1.63 to 0.38; monovision up to − 0.75 D)	− 0.32 ± 0.54 (-1.13 to 0.25; monovision up to − 1.0 D)	0.96	0.36	0.64	0.73
Delta SEQ attempted/achieved	0.20 ± 0.32 (− 0.63 to 0.38)	0.26 ± 0.35 (− 0.88 to 0.38)	0.25 ± 0.47 (− 0.88 to 0.63)	0.40	0.50	0.60	0.96
Target accuracy ≤ ± 0.50 D	18/21 (85.7%)	16/21 (76.2%)	13/21 (61.9%)	0.20	0.43	0.08	0.32
Mean astigmatism postOP (D)	− 0.39 ± 0.26 (− 1.00 to 0.00)	− 0.49 ± 0.26 (− 1.00 to 0.00)	− 0.62 ± 0.32 (− 1.50 to 0.00)	**0.04 **	0.24	**0.016 **	0.15
Asti ≤ ± 0.50 D postOP	19/21 (90.5%)	16/21 (76.2%)	11/21 (52.4%)	**0.02**	0.21	**0.006**	0.11
**UDVA** post (logMAR)	− 0.02 ± 0.06 (− 0.10 to 0.10)	− 0.03 ± 0.14 (− 0.20 to 0.30)	− 0.02 ± 0.11 (− 0.20 to 0.20)	0.95	0.57	0.97	0.66
Plano eyes with 20/20	17/19 (89.5%)	15/17 (88.2%)	16/19 (84.2%)	0.89	0.35	0.63	0.61
**CDVA** post (logMAR)	− 0.07 ± 0.05 (− 0.10 to 0.00)	− 0.10 ± 0.10 (− 0.20 to 0.10)	− 0.06 ± 0.09 (− 0.20 to 0.10)	0.20	0.24	0.85	0.29
Change in CDVA (logMar)	− 0.02 ± 0.05 (− 0.10 to 0.00)	− 0.05 ± 0.08 (− 0.20 to 0.10)	− 0.04 ± 0.07 (− 0.20 to 0.00)	0.79	0.60	0.75	0.85
**Safety Index** (postCDVA/präCDVA)	1.13	1.06	1.11	0.75	0.91	0.63	0.76
**Efficacy Index** (postUDVA/ präCDVA)	1.04 (plano target)	1.03 (plano target)	1.01 (plano target)	0.87	0.86	0.62	0.75

UDVA improved comparably in all groups (flat: − 0.02 ± 0.06 vs. regular: − 0.03 ± 0.14 vs. steep: − 0.02 ± 0.11 D; p = 0.95). The percentage of eyes achieving 20/20 and better UDVA was comparable between all groups (89.5 vs. 88.2 vs. 84.2%; eyes not targeted for plano excluded; p = 0.89). The efficacy index was similar across all groups (1.04 vs. 1.03 vs. 1.01, p = 0.87).

### Keratometric and manifest refractive change

Mean K values decreased from 41.3 ± 0.7D (flat), 45.4 ± 1.0D (regular) and 47.7 ± 0.6D (steep) to 36.8 ± 1.3D, 40.6 ± 1.6D and 42.9 ± 1.0D, respectively (all changes with p < 0.01). The ratio between the surgically induced change in MRSE and the surgically induced change in mean K values was 1.22 (flat) vs. 1.20 (regular) vs. 1.23 (steep) with omnibus p = 0.92. The corresponding Spearman correlation coefficients (Rho) were 0.53 (p < 0.001), 0.59 (p < 0.001) and 0.65 (p < 0.001), respectively.

### Higher order aberrations

Preoperatively, the flat group showed significantly lower values of total HOAs than the two other groups (flat: 0.296 ± 0.072 vs. regular: 0.376 ± 0.119, p = 0.012; vs. steep: 0.428 ± 0.125, p = 0.002; Table 3). This effect seemed to be largely caused by spherical aberration, which also was significantly lower in the flat group (flat: 0.134 ± 0.069 vs. regular: 0.234 ± 0.095, p = 0.0004; vs. steep: 0.290 ± 0.102; p < 0.00001). Coma and trefoil were comparable between all groups (p > 0.054 for all comparisons).

**Table 3:** Changes in higher-order aberrations related to SMILE.

	Flat	Regular	Steep	Omnibus p-value	Post-hoc F–R	Post-hoc F–S	Post-hoc R–S
**HOA preOP (µm)**							
RMS HOA	0.296 ± 0.072	0.376 ± 0.119	0.428 ± 0.125	**0.001**	**0.012**	**0.002**	0.17
Spherical aberration	0.134 ± 0.069	0.234 ± 0.095	0.290 ± 0.102	** < 0.00001**	**0.0004**	** < 0.00001**	0.07
Coma	0.168 ± 0.093	0.196 ± 0.105	0.229 ± 0.107	0.16	0.36	0.054	0.31
Trefoil	0.075 ± 0.052	0.091 ± 0.081	0.062 ± 0.030	0.29	0.46	0.33	0.14
**HOA postOP (µm)**							
RMS HOA	0.572 ± 0.201	0.558 ± 0.175	0.648 ± 0.176	0.61	0.8	0.2	0.11
Spherical aberration	0.308 ± 0.137	0.311 ± 0.144	0.322 ± 0.140	0.95	0.94	0.75	0.82
Coma	0.369 ± 0.204	0.310 ± 0.165	0.425 ± 0.165	0.12	0.31	0.33	0.029
Trefoil	0.109 ± 0.049	0.086 ± 0.053	0.097 ± 0.049	0.33	0.15	0.42	0.43
**Change in HOA (µm)**							
RMS HOA	0.276 ± 0.233	0.182 ± 0.165	0.220 ± 0.178	0.3	0.14	0.39	0.48
Spherical aberration	0.174 ± 0.133	0.077 ± 0.123	0.031 ± 0.110	**0.001**	0.019	**0.0005**	0.21
Coma	0.201 ± 0.240	0.114 ± 0.189	0.196 ± 0.175	0.3	0.2	0.94	0.15
Trefoil	0.034 ± 0.068	− 0.005 ± 0.074	0.034 ± 0.051	0.1	0.08	0.98	0.051

Total HOAs (p = 0.30), coma (p = 0.30) and trefoil (p = 0.10) were induced by SMILE to a comparable extent in all groups. The flat group, however, experienced a significantly higher induction of spherical aberration than the steep group (vs. 0.031 ± 0.110; p = 0.0005), and a tendentially higher induction of spherical aberration than the regular group (0.174 ± 0.133 vs. 0.077 ± 0.123; p = 0.019 not significant for post-hoc testing).

## Discussion

The present study was designed to test the safety, efficacy and higher order aberrometric outcome obtained by myopic SMILE in eyes with extremely flat and steep keratometry readings. Analyzing the outcomes in three matched groups, we concluded that SMILE produced non-inferior results in flat (< 42 D) and steep (≥ 47 D) corneas as compared to the regular control group matched by age, follow-up, MRSE, sphere, astigmatism, CDVA, UDVA and SMILE parameters.

Concerning safety, all groups exhibited an excellent profile. No eye lost two or more lines of CDVA, and only 10% of eyes in the regular group lost one line. Moreover, no ectasia was observed during the mean follow-up of 9.2 months, bearing in mind that eyes with steep keratometry might predispose to, or display a sub-clinical *forme fruste* of keratoconus. Since values between 47.2 and 48.7 D might be regarded as “probable”, and values > 48.7 D as “probably definite” keratokonus^18^, refractive surgery in such eyes requires thorough bilateral ruling-out of pre-existing ectatic disease, e.g. using the Belin-Ambrósio enhanced ectasia screening based on Scheimpflug imaging^19^. Although most case reports published describe ectasia after 6 to 12 months after SMILE, a time frame encompassed in our study, long-term follow-up remains warranted in such steep keratometry after keratorefractive treatment. Conversely, the majority of patients reported with ectasia after SMILE already had suspicious topography and keratometry preoperatively^20^, which was carefully ruled out in every eye included in this study.

Concerning efficacy, both groups with flat and steep corneas showed similar, comparable results in the final MRSE and refractive accuracy as compared to regular keratometry. However, the percentage of patients with postoperative manifest cylinder of < 0.50 D was lower, and the total value of astigmatism was significantly higher in the steep than the flat group. Nevertheless, these subtle, clinically probably irrelevant differences did not translate into differences in visual acuity. In our study, both the flat and steep groups achieved comparable, non-inferior percentages of eyes achieving UDVA of 20/20 or better as compared to the regular group. Although it is unclear whether a larger sample size would have been able to determine, even if subtle, inferiority in astigmatic target accuracy in extremely steep corneal curvature, the non-inferior visual results reported in our study might suggest that any detectable differences might be more of academic, and less of clinical value.

In addition to the standard reporting of refractive outcomes, our study performed an in-depth analysis of HOAs and their relationship to preoperative corneal curvature. Theoretically, discrepancies between the uniform radius of the SMILE contact glass interface and extreme corneal keratometry might result in tissue compression or suction, thus distorting the stroma. This could entail the creation of irregular cap or lenticule interfaces with potential ramifications on iatrogenic HOA induction. Interestingly, neither the flat nor the steep group showed increased HOA values as compared to the regular group, corroborating this hypothesis. Of note, we found that eyes in the flat keratometry group responded to myopic SMILE with significantly more spherical aberration induction than the steep, and tendentially more than the regular group. As stated above, this might result from the forced accommodation of the flat corneal surface into the relatively steeper contact glass, causing irregular lenticule borders within the stroma after tissue relaxation. The clinical consequence of this finding may be negligible as the flat keratometry group exhibited the lowest preoperative level of spherical aberration of all three groups, that was still lowest of all groups postoperatively.

Limitations of this pilot study mainly include its small sample size. Larger studies are warranted to exclude differences not detected in this study with only 21 eyes per group; and moreover, such studies should also be designed with a homogenous structured follow-up with twelve-month data, which our pilot study could not provide as yet (end follow-up between 8 and 10 months per group, which was not statistically significantly different, but might affect outcomes).

In conclusion, this matched comparative study suggests that myopic SMILE also produces safe and effective, non-inferior refractive and visual outcomes in eyes with extremely flat and steep keratometry. In eyes with excessively steep keratometry, utmost caution should be taken to exclude underlying ectatic disease.
